# NEUBOrg: Artificially Induced Pluripotent Stem Cell-Derived Brain Organoid to Model and Study Genetics of Alzheimer’s Disease Progression

**DOI:** 10.3389/fnagi.2021.643889

**Published:** 2021-02-23

**Authors:** Sally Esmail, Wayne R. Danter

**Affiliations:** 123Genetix, London, ON, Canada

**Keywords:** Alzhimer’s disease, brain organoids, stem cell-derived brain organoids, machine learning, genetic risk factor, drug discovery, biomarkers

## Abstract

Alzheimer’s disease (AD) is the most common type of neurodegenerative diseases. There are over 44 million people living with the disease worldwide. While there are currently no effective treatments for AD, induced pluripotent stem cell-derived brain organoids have the potential to provide a better understanding of Alzheimer’s pathogenesis. Nevertheless, developing brain organoid models is expensive, time consuming and often does not reflect disease progression. Using accurate and inexpensive computer simulations of human brain organoids can overcome the current limitations. Induced whole brain organoids (aiWBO) will greatly expand our ability to model AD and can guide wet lab research. In this study, we have successfully developed and validated artificially induced a whole brain organoid platform (NEUBOrg) using our previously validated machine learning platform, DeepNEU (v6.1). Using NEUBorg platform, we have generated aiWBO simulations of AD and provided a novel approach to test genetic risk factors associated with AD progression and pathogenesis.

## Introduction

The modern era of human stem cell research was launched with a publication by Professor Yamanaka’s group in 2007 (Takahashi et al., 2007). This landmark paper demonstrated conclusively that human fibroblasts could be transformed, with four transcription factors and optimal conditions, into cells closely resembling human pluripotent stem cells (hPSC). These transformed cells have become widely referred to as induced pluripotent stem cells (iPSC). Since 2007 iPSCs have become a mainstay technology for disease modeling, stem cell therapies, drug discovery and specific cell line differentiation. More recently iPSCs have been used to develop tissue specific spheroids and more complex organoids show some early promise both as research tools and disease specific transplant options (Hattori, 2014; Lancaster and Knoblich, 2014; Lancaster and Huch, 2019). An organoid has been defined as an artificially grown mass of cells or tissue that resembles an organ. While still in its infancy, the science of human organoids has been used successfully to develop multiple organoid types including intestine, heart, pancreas, liver, lung, and brain to name a few (Kim et al., 2020).

Cerebral organoids have been produced with some early successes (Hattori, 2014; Lancaster and Knoblich, 2014). Cerebral organoids continue to evolve as useful tools for modeling more common chronic and degenerative neurologic diseases like Alzheimer’s disease, Parkinson’s disease, and rarer neurological disorders like RETT syndrome, Huntington’s disease and Zika microcephaly are also amenable to cerebral organoid modeling (Logan et al., 2011; Chen et al., 2019). In general, any neurologic disease that can be represented in an induced stem cell model can theoretically also be represented in a cerebral organoid. To date, two main approaches have been employed to produce cerebral organoids from human stem cells (Qian et al., 2019). These two methods have been labeled as unguided and guided. The unguided approach relies on the tendency of iPSC to develop toward neural precursors if given enough time and an optimal cellular environment. The guided method uses sequential combinations of small molecules and modified media to generate cerebral organoids. Both methods produce organoids that take many months to mature, result in recognizable and semi-organized cerebral tissue with similar limitations. Growing cerebral organoids *in vitro* has been challenging in several important ways. For example, modeling cerebral vasculature and a functional blood brain barrier remain problematic (Zhao et al., 2015; Lancaster and Huch, 2019; Qian et al., 2019; Marx, 2020; Workman and Svendsen, 2020). In addition, the *in vitro* organoids lack a blood supply and this important deficit results in a variable amount of central necrosis as the organoids outgrow the ability of diffusion to deliver oxygen, nutrients, and remove toxic waste by products (Wang, 2018).

More complex whole brain organoids represent a logical advancement of cerebral organoids. This technology attempts to reproduce forebrain, midbrain, and hindbrain structures with rostral-caudal as well as ventral-dorsal organization. The development of whole brain organoids has been advanced by combining regionally specific organoids *in vitro* like cerebral, cerebellar, retinal organoids, and directing the development of functional vascular elements (Chen et al., 2020). Rotating bioreactors with complex media have provided additional improvement in evolving whole brain organoids (Qian et al., 2018). Overall, this process is technologically demanding and requires considerable time and expense to achieve modest results. A recent study has concluded that at present, whole brain organoids do not faithfully reproduce all elements of the human brain (Bhaduri et al., 2020). This is particularly true regarding the internal architecture and neural connections. One suggested explanation for the observed differences could be the significant and multiple stresses that occur during the growth and development of iPSC and avascular organoids over protracted periods of time. This suggestion is supported by the observation that transplanted brain organoids tend to become more organized and representative of the native neural tissue (Bhaduri et al., 2020).

We believe that the future for whole brain organoid research including novel therapeutic possibilities is quite promising and to date remarkable progress has been achieved (Kim et al., 2020). Given the considerable cost and limitations of current *in vitro* derived whole brain organoids we also believe that validated, easily customizable and relatively inexpensive computer simulations of artificially induced whole brain organoids (aiWBO) could represent an important step forward. Such a technology could be an important tool for simulating many neurological diseases and lead to important new therapeutic insights to guide wet lab research in a timely and cost-effective manner.

### Modeling Alzheimer Disease Using iPSC-Derived Brain Cells

Alzheimer’s disease (AD) is the most common chronic neurodegenerative disease characterized by progressive loss of cognition and disruption of basic functions, such as swallowing, walking, attention, and memory (Alzheimer’s Association, 2019). AD is the sixth leading cause of death in the United States and the fifth leading cause of death among seniors (Alzheimer’s Association, 2020). Increasing evidence has suggested that loss or dysfunction of different brain cell types likely contribute to AD progression (De Strooper and Karran, 2016). Thus, modeling these cells and examining their interactions will shed mechanistic insights into AD pathogenesis. Accordingly, significant efforts have been put to building models that incorporate multiple iPSC-derived brain cell types. However, the ability to obtain iPSC-derived brain cells that mimic human brain remains a significant challenge. Validated whole brain organoid simulations could represent an important tool for enabling neurodegenerative disease modeling, biomarker identification and drug discovery.

The main purpose of this project was to extend our previous DeepNEU research (Danter, 2019; Esmail and Danter, 2019, 2020) by developing and validating aiWBO using the latest validated version of the DeepNEU (v6.1) machine learning platform. In this study, the DeepNEU platform was used to generate aiWBO simulations of AD. To our knowledge this is the first time that a simulation of a Whole Brain Organoid has been attempted. These aiWBO simulations have been named NEUBOrg.

## Materials and Methods

The DeepNEU stem cell simulation platform is a literature validated hybrid deep-machine learning system with elements of fully connected recurrent neural networks (RNN), cognitive maps (CM), support vector machines (SVM), and evolutionary systems (GA). The detailed methodology for simulation development and validation plus the description of the evolving DeepNEU database used in these experiments is described in detail in Supplementary Appendix 1 and previously in Danter (2019), Esmail and Danter (2019, 2020).

The current DeepNEU database (v6.1) contains the information found in the previous version (v5.0) plus an important information upgrade in the form of new genotypic concepts, phenotypic concepts and relationships. Most of these new organoid concepts and relationships were used to generate signatures for identifying specific cell types, brain regions, cortical layers, the blood brain barrier, provide evidence of a rudimentary circulation and cellular stresses. For example, the previous DeepNEU database (v5.0) contained 4,206 gene/proteins or phenotypic concepts and 37,223 non-zero relationships while the current version (6.1) contains 4,516 gene/proteins or phenotypic concepts and 41,493 non-zero relationships. This represents more than 4,700 new relationships specifically relevant to developing whole brain organoids. Each gene/protein and phenotypic concept in DeepNEU (v6.1) has on average more than nine gene/protein or phenotypic inputs and outputs.

### The DeepNEU Simulations

#### The NEUBOrg Whole Brain Organoid (aiWBO) Simulations

The main purpose of this project was to extend our previous DeepNEU research by developing and validating a whole brain organoid simulation (aiWBO) using the latest version of the DeepNEU (v6.1) machine learning platform. To accomplish this, we used an approach similar to that described by Takahashi et al. (2007) to transform human fibroblasts into induced pluripotent stems cells (iPSC). Several important modifications to the original 2007 protocol were required to promote differentiation from aiPSC to aiWBO. The modified protocol began with the activation of four transcription factors OCT4, cMYC, KLF4, and SOX2. In addition, we used a simulated B27 neural media with supplementary zinc and ascorbic acid in the presence of doxycycline and normal levels of ambient oxygen (21%) and increased carbon dioxide (5%). The aiPSC to aiWBO transformation using the above cocktail was carried out in a simulated slow rotating low shear bioreactor, which has been shown to improve the production of WBO from iPSCs. A bioreactor is a vessel that creates an optimal environment that facilitates biological reactions and is used to culture aerobic cells and organoids. The simulated bioreactor environment used in this project was based on protocols for organoid development described in detail elsewhere (Lancaster and Knoblich, 2014; Qian et al., 2018). For all simulations age was fixed at 65 years. AGE is an input value that varies between −1 and +1 and can be set by the experimenter or evolved by the simulation. A value of −1 represents time of birth, 0 represents 50 years and +1 represents an age ≥ 100 years of age. All aiWBO simulation experiments were carried out in triplicate. The complete cocktail is summarized below in Table 1.

**TABLE 1:** Unguided aiPSC to aiWBO simulation cocktail.

Cocktail	Components (simulated)
Yamanaka (2007) transcription factors	OCT4, cMYC, KLF4, and SOX2 turned ON
B27 neural media	Biotin, amino acids, ascorbate, catalase, cortisol, FGF2/bFGF, glutathione, albumin, insulin, SOD1 (Cu/Zn), MnSOD/SOD2, progesterone, retinol/vitamin A, thyroid hormones (T3/T4), transferrin, VitE/tocopherol, L-carnitine locked ON
Supplements	Zinc and Doxycycline locked ON
Rotating bioreactor environment (optimized)	B27 media + [CO_2_] = 5%, [O_2_] = 21%, glucose, temperature = 37 degrees C locked ON and High shear forces locked OFF
Age- (65 years of age locked ON)	57 input factors, 29 positive, and 28 negative

The aiWBO were designed to simulate the (i) diverse neural cell types, (ii) rostral caudal brain regions, (iii) ventral dorsal regions where possible and the (iv) six horizontal layers of the cerebral cortex, and (v) the four layers of the cerebellar cortex. The spinal cord was not simulated in the current project. The main cell types included are neural precursor cells (NPC/NSC) found primarily in the radial glial (RG) layer, neurons, astrocytes, oligodendrocyte precursors (OPC), oligodendrocytes, interneurons, and microglial cells. Additional cell types include endothelial cells and pericytes which are important components of the blood brain barrier (BBB). The rostral caudal regions simulated include forebrain, midbrain and hindbrain. The ventral dorsal regions include ventral forebrain, etc. The horizontal layers of the cerebral cortex arranged from outer to inner include Layers 1–6 and the 4 main layers of the cerebellar cortex from outer to inner are also simulated. NEUBOrg platform, aiWBO simulations, was built based on the following comprehensive literature to provide genotypic and phenotypic markers for identifying cell types, brain regions and cortical layers (Ahn et al., 2004; Logan et al., 2011, 2020; Santos et al., 2011; Kirsch et al., 2012; Hattori, 2014; Lancaster and Knoblich, 2014; Lippmann et al., 2015; Zemke et al., 2015; Zhao et al., 2015; Tkachenko et al., 2016; Espuny-Camacho et al., 2018; Krefft et al., 2018; McKenzie et al., 2018; Qian et al., 2018, 2019; Wang, 2018; Chen et al., 2019, 2020; Do and Hong, 2019; Gerakis and Hetz, 2019; Kim et al., 2019, 2020; Lancaster and Huch, 2019; Velasco et al., 2019; Bhaduri et al., 2020; Papaspyropoulos et al., 2020). A compilation of relevant markers is presented in Supplementary Tables S1–S5.

#### The NEUBOrg Whole Brain Organoid Simulations Applied to Alzheimer’s Disease

Once validated, the unguided aiWBO were used to simulate a whole brain organoid affected by Alzheimer’s disease. To accomplish the disease simulations (aiWBO-APOE4) two concepts were modified. First, APOE4 was locked ON to simulate an APOE4 duplication/GOF mutation. To simulate an important inhibitory effect of an APOE4 mutation, beta amyloid clearance was locked OFF. The cocktail and process were otherwise identical to that outlined above for the developing the wild type aiWBO simulations. All aiWBO-APOE4 (AD) simulation experiments were carried out in triplicate.

A detailed literature review of genotypic and phenotypic features was used to develop a profile of Alzheimer’s Disease that we used to evaluate the performance of the AD simulations compared to the aiWBO (Romito-DiGiacomo et al., 2007; Santos et al., 2011; Raja et al., 2016; Arbor, 2017; Gerakis and Hetz, 2019; Madadi et al., 2019; Brighi et al., 2020; Papaspyropoulos et al., 2020; Ribeiro et al., 2020; Silva et al., 2020). Negative inputs inhibit the feature while positive inputs promote the feature. A compilation of the large number and status of inputs that constitute the AD feature profile is presented in Table 2 below.

**TABLE 2:** Alzheimer’s Disease feature profile.

Alzheimer’s Disease (AD) features (*N* = 10)	Genotypic/phenotypic feature inputs
Amyloid beta plaques	*N* = 9, 3 negatives + 6 positives
Amyloid beta protein (42/40) clearance	*N* = 51, 23 negatives + 28 positives
Amyloid beta protein (Ab1-42/Ab1-40)	*N* = 47, 24 negatives + 23 positives
Amyloid-beta-oligomers/aggregation	*N* = 45, 23 negatives + 22 positives
ApoE4 (>ApoE3)	*N* = 17, 8 negatives + 9 positives
APP/amyloid precursor protein	*N* = 50, 24 negatives + 26 positives
NFTs/neurofibrillary tangles	*N* = 10, 4 negatives + 6 positives
Tau proteins/MAPT	*N* = 27, 16 negatives + 11 positives
Tau protein phosphorylated	*N* = 52, 25 negatives + 27 positives
Tau protein aggregation	*N* = 10, 3 negatives + 7 positives

We also analyzed the aiWBO simulation data for evidence of cellular stress and neuron death that have commonly been observed in organoids and is thought to impair organoid development (Bhaduri et al., 2020). The same paper was used to develop a genotypic and phenotypic features profile of organoid cellular stress and neuron cell death. Negative inputs inhibit the feature while positive inputs promote the feature. A compilation of the inputs that constitute a cell stress and neuron cell death profile is presented in Table 3 below.

**TABLE 3:** Cell stress and neuron cell death feature profile.

Cell stress and neuronal cell death features (*N* = 6)	Genotypic/phenotypic feature inputs
Glycolysis	*N* = 51, 19 negatives + 32 positives
PGK1	*N* = 6, 0 negatives + 6 positives
ER_Stress	*N* = 84, 29 negatives + 55 positives
ARCN1	*N* = 1, 0 negatives + 1 positive
GORASP2	*N* = 1, 0 negatives + 1 positive
Neuron cell death	*N* = 43, 10 negatives + 33 positives

#### DeepNEU Platform Statistical Analysis

Consistent with previously projects, the statistical analysis of all aiWBO and aiWBO-APOE4 predictions vs. the published and previously unseen wet lab data used the unbiased binomial test. This test provides an exact probability, can compensate for prediction bias, and is ideal for determining the statistical significance of experimental deviations from an actual distribution of observations that fall into two outcome categories (e.g., success or fail or agree or disagree). For all aiWBO simulations, the null hypothesis (H_0_) states that there is no significant relationship between simulation predictions and published wet lab data. The H_0_ is rejected if the *p*-value is <0.05 and is interpreted to indicate that the observed relationship between aiWBO predictions and actual outcomes is unlikely to have occurred by chance alone. For other between group (e.g., aiWBO vs. aiWBO-APOE4) comparisons, the Mann-Whitney *U* test of significance was used (Addinsoft, 2019). This non-parametric test was chosen because some of the data was not normally distributed.

## Results

### The DeepNEU Platform Specification

The current DeepNEU database (v6.1) contains 4,516 gene/protein or phenotypic concepts and 41,493 non-zero relationships resulting in a large amount of information flowing into and out of each node in the fully connected recurrent network. On average, each node in the network initially has >9 inputs and >9 outputs. An updated analysis of all positive and negative network connections revealed a bias toward positive outputs. The pretest probability of a positive outcome prediction is 0.656 and the pretest probability of a negative prediction is therefore 0.344. This system bias was used when applying the binomial test to all simulation outcomes.

### The aiWBO Wild Type Simulations

The unsupervised whole brain organoid (aiWBO) simulations converged quickly (45 iterations) to a new system wide steady state without evidence of overtraining after 1,000 iterations.

#### Neural Cell Types

**FIGURE 1:**
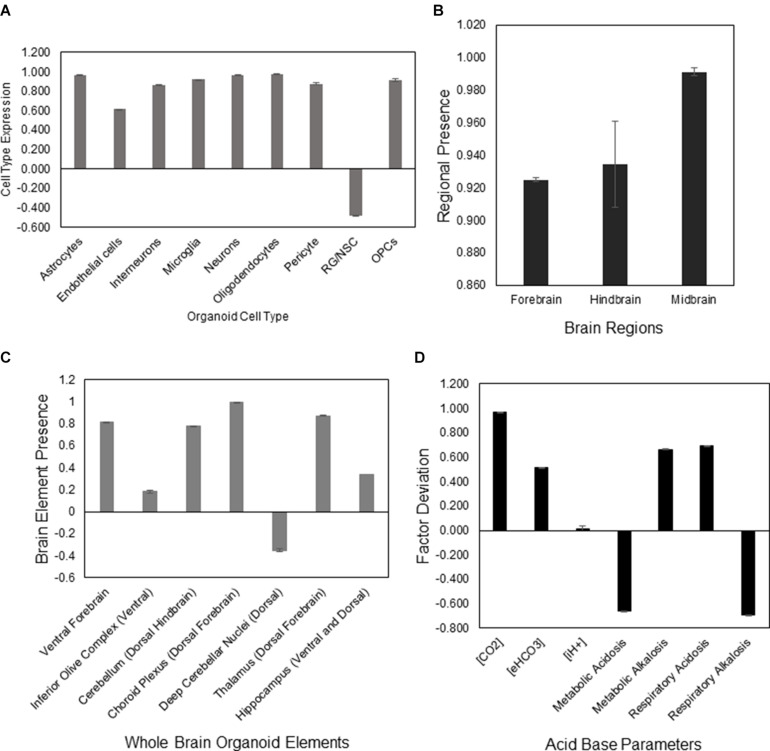
DeepNEU simulation of differentiated WT aiWBO cell types, regions, elements, and acid base status. **(A)** Expression of basic aiWBO cell types based on genotypic markers. **(B)** Presence of the three aiWBO brain regions based on genotypic markers. **(C)** Presence of the ventral and dorsal aiWBO brain elements based on genotypic markers. **(D)** The acid base status of the aiWBO based on phenotypic factors. The vertical y-axes represents the semiquantitative levels of concepts that are estimated by DeepNEU regarding an arbitrary base line where 0 = base line, 1 = maximum expression or presence, and −1 = minimum expression level or presence. The horizontal x-axes represent the individual aiWBO concepts being simulated. Data represent mean of three experiments ±95% confidence interval.

The aiWBO correctly predicted the expression of all nine neural cell types commonly found in human brain organoids. The probability that all (*N* = 9) cell type outcomes were predicted by chance alone using the binomial test is 0.023. These results are summarized in Figure 1A.

#### Rostral-Caudal Brain Regions

The aiWBO correctly predicted the expression of all three regions commonly found in human brain organoids. These regions are Forebrain, Midbrain and Hindbrain. The spinal cord was not simulated in the current version. The probability that the expression of all (*N* = 3) regions were predicted by chance alone using the binomial test is 0.282. These results are summarized in Figure 1B.

#### Ventral-Dorsal Brain Regions

The aiWBO correctly predicted the expression of all seven ventral (anterior)-dorsal (posterior) regions commonly found in human brain organoids. The probability that the expression of all (*N* = 7) regions were predicted by chance alone using the binomial test is 0.052. These results are summarized in Figure 1C.

#### Acid-Base Status of the Organoid

The aiWBO predicted the expression of all seven concepts representative of a mixed (compensated) metabolic-alkalosis and respiratory acidosis in human brain organoids. The probability that the expression of all (*N* = 7) concepts associated with the expression of a compensated metabolic-alkalosis and respiratory acidosis were predicted by chance alone using the binomial test is 0.052. These results are summarized in Figure 1D.

#### Cerebral Cortical Layers

**FIGURE 2:**
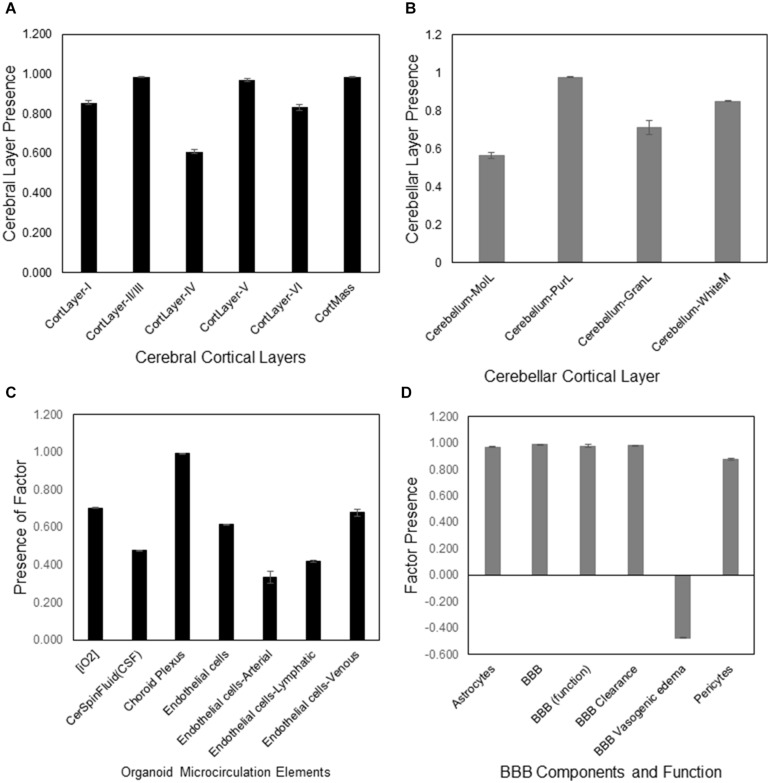
DeepNEU simulation of cerebral cortical layers, cerebellar cortical layers, microcirculation and BBB of WT aiWBO. **(A)** Presence of the six aiWBO cerebral cortical layers based on genotypic markers. An estimate of cortical mass is derived by combining the six cortical layers. **(B)** Presence of the four cerebellar layers based on genotypic markers. **(C)** Presence of aiWBO microcirculation elements based on phenotypic markers. **(D)** The presence of aiWBO BBB elements and function based on phenotypic factors. The vertical y-axes represent the semiquantitative levels of concepts that are estimated by DeepNEU regarding an arbitrary base line where 0 = base line, 1 = maximum expression or presence, and −1 = minimal expression level or presence. The horizontal x-axes represent the individual aiWBO concepts being simulated. Data represent mean of three experiments ±95% confidence interval.

The aiWBO correctly predicted the expression of all six cerebral cortical layers commonly found in human brain organoids. The probability that the expression of all (*N* = 6) cerebral cortical layers were predicted by chance alone using the binomial test is 0.08. These results are summarized in Figure 2A.

#### Cerebellar Cortical Layers

The aiWBO correctly predicted the expression of all six cerebellar layers cortical commonly found in human brain organoids. The probability that the expression of all (*N* = 4) cerebral cortical layers were predicted by chance alone using the binomial test is 0.185. These results are summarized in Figure 2B.

#### Microcirculation

The aiWBO correctly predicted the expression of all seven concepts representative of a microcirculation in human brain organoids. The probability that the expression of all (*N* = 7) concepts associated with the expression of a microcirculation were predicted by chance alone using the binomial test is 0.052. These results are summarized in Figure 2C.

#### Blood Brain Barrier (BBB)

The aiWBO correctly predicted the expression of all six concepts representative of a BBB in human brain organoids. The probability that the expression of all (*N* = 6) concepts associated with the expression of a BBB were predicted by chance alone using the binomial test is 0.185. These results are summarized in Figure 2D.

#### Cellular Stress and Neuron Cell Death

The aiWBO simulations predicted the expression of six markers of cell stresses and neuron cell death commonly found in *in vitro* human brain organoids consistent with published data (Bhaduri et al., 2020). These results are summarized in Figure 5A.

**FIGURE 3:**
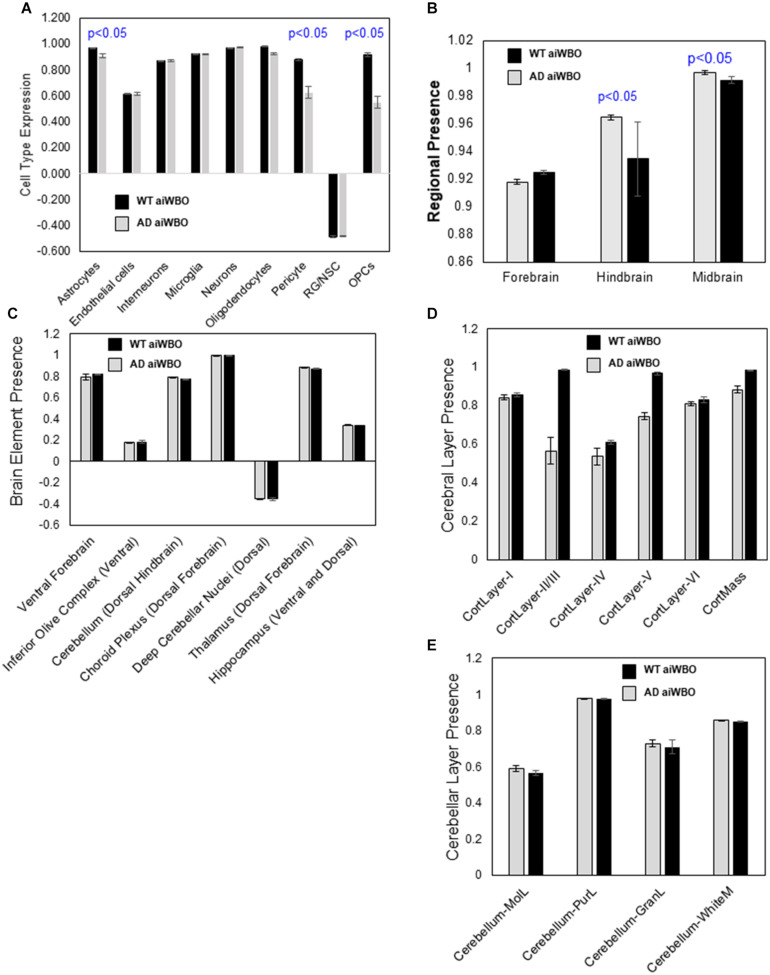
Comparison of aiWBO-WT vs. aiWBO-AD simulation results. **(A)** Comparison of aiWBO simulations of WT vs. AD cell types. **(B)** Comparison of brain region concepts in WT vs. AD simulations. **(C)** Comparison of ventral-dorsal concepts in WT vs. AD simulations. **(D)** Comparison of cerebral cortical layers in WT vs. AD simulations. **(E)** Comparison of cerebellar cortical layers in WT and AD simulations. The vertical y-axes represent the semiquantitative levels of concepts that are estimated by DeepNEU regarding an arbitrary base line where 0 = base line, 1 = maximum expression or presence, and −1 = minimal expression level or presence. The horizontal x-axes represent the individual aiWBO concepts being simulated. Data represent mean of three experiments ± 95% confidence interval. All *p*-values from Mann-Whitney *U* test.

**FIGURE 4:**
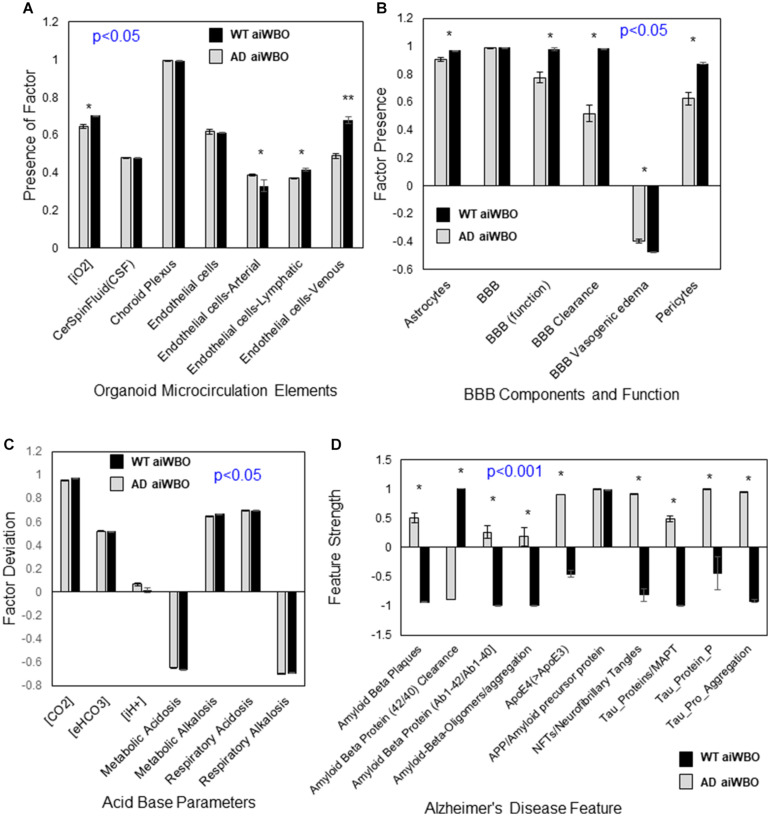
Comparison of aiWBO-WT vs. aiWBO-AD simulation results for microcirculation, BBB, Acid-Base status, and Alzheimer’s Disease (AD) features. **(A)** Comparison of microcirculation elements in WT vs. AD simulations. **(B)** Comparison of BBB concepts in WT vs. AD simulations. **(C)** Comparison of acid base concepts in WT vs. AD simulations. **(D)** Comparison of Alzheimer’s Disease features in WT vs. AD simulations. The vertical y-axes represent the semiquantitative levels of concepts that are estimated by DeepNEU regarding an arbitrary base line where 0 = base line, 1 = maximum expression or presence, and −1 = minimal expression level or presence. The horizontal x-axes represent the individual aiWBO concepts being simulated. Data represent mean of three experiments ± 95% confidence interval. All *p*-values from Mann-Whitney *U* test. *p < 0.05.

**FIGURE 5:**
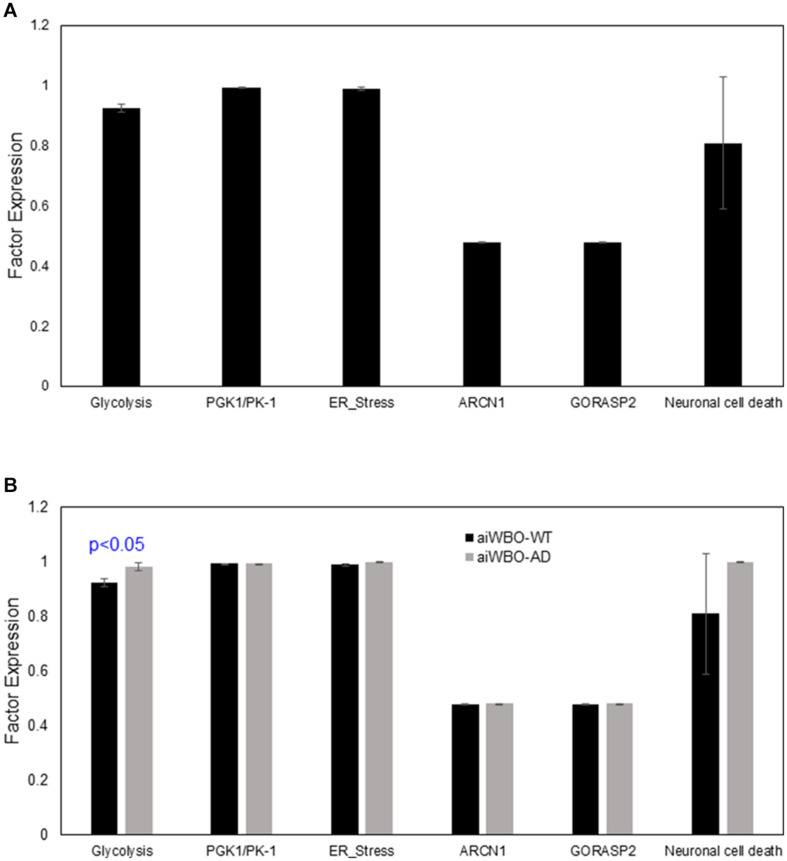
Comparison of aiWBO-WT vs. aiWBO-AD simulation prediction of cellular stress and neuronal cell death. **(A)** Simulation prediction of cellular stress and neuronal cell death in aiWBO-WT. **(B)** Comparative simulation predictions of cellular stress and neuronal cell death in aiWBO-WT vs. aiWBO-AD. The vertical y-axes represent the semiquantitative levels of concepts that are estimated by DeepNEU regarding an arbitrary base line where 0 = base line, 1 = maximum expression or presence, and −1 = minimal expression level or presence. The horizontal x-axes represent the individual aiWBO concepts being simulated. Data represent mean of three experiments ± 95% confidence interval. All *p*-values from Mann-Whitney *U* test.

### Summary Results for aiWBO Simulations

Taken together the aiWBO correctly predicted the expression of 48 elements consistent with a pattern seen in a whole brain organoid and seven elements consistent with the presence of a compensated metabolic-alkalosis and respiratory acidosis. The probability that the expression of all (*N* = 55) concepts were predicted by chance alone using the binomial test is <0.00000001.

#### The aiWBO-APOE4 Alzheimer’s Disease (AD) Simulations

The unsupervised whole brain organoid (aiWBO-APOE4) AD simulations converged quickly (42 iterations) to a new system wide steady state without evidence of overtraining after 1,000 iterations.

#### Neural Cell Types

The aiWBO-APOE4 (AD) simulations correctly predicted the expression of all nine neural cell types commonly found in human brain organoids. The probability that all (*N* = 9) cell type outcomes were predicted by chance alone using the binomial test is 0.023. A statistical analysis using the Mann-Whitney *U* test indicated that there were significant decreases in the expression of astrocytes, pericytes, OPCs, and oligodendrocytes with the AD simulations (*p* < 0.01). We also observed a marginal increase in RG/NSC (*P* < 0.05). All other cell types including neurons were not significantly different between aiWBO and AD simulations. These results are summarized in Figure 3A.

While the major cell target of advanced AD is neurons, deleterious effects on synapses are among the earliest pathologic changes. When we examined the impact of increased APOE4 on the AD simulations using a 7-element, literature validated profile, the effects were largely consistent with significant decline in synaptogenesis and synaptic function. These results are summarized in Supplementary Table S6.

#### Rostral-Caudal Brain Regions

The aiWBO-APOE4 (AD) simulations correctly predicted the expression of all three regions commonly found in human brain organoids. These regions are Forebrain, Midbrain and Hindbrain. The spinal cord was not simulated in the current version. The probability that the expression of all (*N* = 3) regions were predicted by chance alone using the binomial test is 0.282. A statistical analysis using the Mann-Whitney *U* test indicated that there was a small but significant (*p* < 0.05) decrease in the expression of the Forebrain and small but significant increase in the Midbrain while the Hindbrain was unchanged in the AD simulations. These results are summarized in Figure 3B.

#### Ventral-Dorsal Brain Regions

The aiWBO-APOE4 (AD) simulations correctly predicted the expression of all seven ventral (anterior)-dorsal (posterior) representative regions commonly found in human brain organoids. The probability that the expression of all (*N* = 7) regions were predicted by chance alone using the binomial test is 0.052. A statistical analysis using the Mann-Whitney *U* test indicated that there were no significant differences (*p* > 0.05) in the expression of all Ventral-Dorsal brain regions in the aiWBO and AD simulations. These results are summarized in Figure 3C.

#### Cerebral Cortical Layers

The aiWBO-APOE4 (AD) simulations correctly predicted the expression of all six cerebral cortical layers commonly found in human brain organoids. The probability that the expression of all (*N* = 6) cerebral cortical layers were predicted by chance alone using the binomial test is 0.08. A statistical analysis using the Mann-Whitney *U* test indicated that there was a significant decrease (*p* < 0.01) in the expression of the Layers 2–5 with a very small but still significant decrease (*p* < 0.05) Layer 6. Layer 1 was unchanged in the AD simulations. Overall, there was also a significant decrease in cerebral cortical mass in AD simulations. These results are summarized in Figure 3D.

#### Cerebellar Cortical Layers

The aiWBO-APOE4 (AD) simulations correctly predicted the expression of all four cerebellar cortical layers commonly found in human brain organoids. The probability that the expression of all (*N* = 4) cerebral cortical layers were predicted by chance alone using the binomial test is 0.185. A statistical analysis using the Mann-Whitney *U* test indicated that there were no significant differences (*p* > 0.05) between expression of all cerebellar cortical layers in the aiWBO and AD simulations. These results are summarized in Figure 3E.

#### Microcirculation

The aiWBO-APOE4 (AD) simulations correctly predicted the expression of all seven concepts representative of a microcirculation in human brain organoids. The probability that the expression of all (*N* = 7) concepts associated with the expression of a microcirculation were predicted by chance alone using the binomial test is 0.052. A statistical analysis using the Mann-Whitney *U* test indicated that there were significant decreases (*p* < 0.05) in the endothelial-arterial, venous and lymphatic components of the Microcirculation in the AD simulations. In addition, the intracellular O_2_ concentration is significantly decreased (*p* < 0.05) in the AD simulations consistent with a degree of microcirculation impairment. These results are summarized in Figure4A.

#### Blood Brain Barrier (BBB)

The aiWBO-APOE4 (AD) correctly predicted the expression of all six concepts representative of a BBB in human brain organoids. The probability that the expression of all (*N* = 6) concepts associated with the expression of a BBB were predicted by chance alone using the binomial test is 0.185. A statistical analysis using the Mann-Whitney *U* test indicated that there were significant decreases in the astrocyte (*p* < 0.05) and pericyte (*p* < 0.01) components of the BBB. In addition, the function of the BBB was significantly decreased (*p* < 0.01) in the AD simulations while the expression of the BBB itself was not different. These results are summarized in Figure 4B.

#### Acid-Base Status of the Organoid

The aiWBO-APOE4 (AD) simulations predicted the expression of all seven concepts representative of a mixed (compensated) metabolic-alkalosis and respiratory acidosis in human brain organoids. The probability that the expression of all (*N* = 7) concepts associated with the expression of a compensated metabolic-alkalosis and respiratory acidosis were predicted by chance alone using the binomial test is 0.052. A statistical analysis using the Mann-Whitney *U* test indicated that there were significant changes (*p* < 0.05) in all components of the AD simulations compared with aiWBO. Most notable there is a significant increase in intracellular H + concentration. Taken together these data suggest a somewhat less well-compensated mixed metabolic alkalosis- respiratory acidosis in the AD simulations. The persistent high CO_2_ concentration is also consistent with the increased CO_2_ concentration (5%) that is part of the cocktail used in generating aiWBO and AD simulations. These results are summarized in Figure 4C.

#### The aiWBO-APOE4 Alzheimer’s Disease (AD) Simulations

The aiWBO-APOE4 simulations predicted the expression of all 10 concepts, representative of the Alzheimer’s Disease (AD) disease profile, in a simulated human brain organoid. The probability that the expression of all (*N* = 10) AD concepts were predicted by chance alone using the binomial test is 0.015. A statistical analysis using the Mann-Whitney *U* test indicated that there were significant changes (*p* < 0.001) in all components of the simulated AD disease profile, except for the expression of amyloid precursor protein (APP) when compared with aiWBO. These results are summarized in Figure 4D.

#### Cellular Stress and Neuron Cell Death

The aiWBO-APOE4 predicted the expression of six markers of cell stresses and neuronal cell death commonly found in *in vitro* human brain organoids. A statistical analysis using the Mann-Whitney *U* test indicated that there were statistically significant increases (*p* < 0.01) in all components of the cell stress profile in the AD disease simulations compared with aiWBO-WT simulations including neuronal cell death. The largest observed difference was an increase in glycolysis. The comparative results are summarized in Figure 5B.

### Summary of AD Simulations

While the AD simulations produced some significant variability when compared with the aiWBO simulation outputs, the AD simulations also correctly predicted the expression of 48 elements consistent with a pattern seen in a whole brain organoid and seven elements suggesting the expression of a compensated metabolic alkalosis and respiratory acidosis. The probability that the expression of all (*N* = 55) concepts were predicted by chance alone using the binomial test is <0.00000001. In addition, there was evidence of increased cellular stress and neuron cell death in the AD simulations relative to aiWBO-WT simulations.

## Discussion

The main purpose of this project was to extend our previous research (Danter, 2019; Esmail and Danter, 2019, 2020) by first developing and then validating a whole brain organoid simulation (aiWBO) using the latest version of the DeepNEU (v6.1) machine learning platform. Our unguided approach toward brain organoid development relied on the natural tendency of iPSC to differentiate toward complex neural outcomes given enough time and optimal physical and environmental conditions. When we evaluated the aiWBO simulations by applying the wet lab, published genotypic and phenotypic features of a whole brain organoid, the results confirm that the simulations performed well in that they reliably reproduced the wet lab profile. The probability that the expression of all (*N* = 46) features were predicted by chance alone using the binomial test is <0.00000001. The important anatomical and regional aspects of the neonatal brain were all predicted accurately. In addition, the simulations appear to have evolved rudimentary elements of a functioning microcirculation and blood brain barrier. Both elements are limited by the absence of a functioning systemic and cerebral circulation. Importantly, the current level of sophistication of these aiWBO simulations has been achieved without expensive or demanding and time-consuming protocols or the need to grow and then combine individual organoids like cerebral, cerebellar and blood brain barrier into more complex whole brain like structures.

Fortunately, the concepts necessary to evaluate acid base status have always been a core capability of the DeepNEU platform. This allowed us to evaluate the status of these artificial whole brain organoid simulations. Overall, the pattern observed at stabilization was that of a compensated or combined metabolic alkalosis and respiratory acidosis. Importantly, the intracellular H+ ion concentration [iH+] deviation from the arbitrary base line is minimal. The high bicarbonate and CO_2_ levels round out the mixed profile. Finally, the organoid appears to be adequately oxygenated. At least part of this complex picture is the result of the normal ambient Oxygen level (21%) and increased CO_2_ (5%) level used in the bioreactor protocol.

Once the aiWBO simulations were validated against the current literature, the DeepNEU platform was be used to generate aiWBO simulations of Alzheimer’s disease (AD), an important and increasingly common chronic degenerative neurologic disease. As outlined above in Table 3, a literature derived 10 feature genotypic and phenotypic AD profile was used to evaluate simulation predictions. When we compared the aiWBO simulation outputs with the AD simulations and allowing for disease related effects, both simulations accurately reproduced the feature profile consistent with the pattern seen in a whole brain organoid. To our knowledge this is the first time that a whole brain organoid simulation of Alzheimer’s Disease (AD) has been attempted.

Alzheimer’s Disease (AD) is known to impact many neural cell types including neurons, oligodendrocytes, astrocytes and microglia (Ref). While more advanced disease largely results in neuronal loss, the current results would suggest that the AD simulations represent an early/milder form of the disease. This conclusion is supported by several other factors. Firstly, Age was arbitrarily set at 65 years for the simulations. Based on the available data this age is widely suggested as a reasonable lower limit for late onset AD (LOAD) and above the upper limit for early onset AD (EOAD). In addition, we wanted to explore the impact of an isolated increase in APOE4, so other factors which are known to exacerbate the disease including diabetes, tobacco abuse, obesity, hypertension, dyslipidemia, and advanced age were all turned off during simulation generation. Finally, this conclusion is supported by the prediction of a significant decline in new synapses formation and loss of synaptic function in AD simulations. Taken together these results are all consistent with an early or milder form of the disease.

In order to create more anatomically correct (3D) whole brain simulations, we evaluated a limited number of (1) Rostral-Caudal features, (2) Ventral-Dorsal features, (3) Cerebral Cortical layers, and (4) Cerebellar Cortical layers. In contrast to wet lab whole brain organoids which are variably disorganized, the aiWBO and aiWBO-APOE3 simulations accurately reproduced the basic anatomical organization of human brain regions and cortical layers. We believe that while encouraging, these anatomic results are rudimentary since validated markers for many brain regions are inadequate or unavailable in the current peer reviewed literature. It is certain this will change as new information is published. Importantly, new information can easily be added to the DeepNEU database in real time as it becomes available.

Regarding Alzheimer’s disease, the current data indicate that the relatively mild disease has small but significant effects on Rostral-Caudal elements but no significant changes in Ventral-Dorsal features. Similarly, there were no significant changes in cerebellar cortical layers. In contrast, there were significant changes in cerebral cortical layers 2–5 and less so in Layer 6. No significant changes were detected in Layer 1. The aiWBO-APOE4 (AD) simulation results are generally like published wet lab results and consistent with early but progressive disease. Pathology has previously indicated that Layers 2–4 tend to be affected early while layers 5–6 are affected later and Layer 1 is unaffected.

Most previous attempts to create whole brain organoids have met with limited success with regards to producing a rudimentary microcirculation. Although the organoids are not connected to a functioning cardiovascular system, the presence of a cerebral microcirculation has important implications as a critical element of a functioning blood brain barrier. As summarized in Figure 4, several important elements of a rudimentary microcirculation were present in the wild type aiWBO simulations. Furthermore, it appears that the simulated microcirculation may be impaired in the AD simulations consistent with known effects patients with AD.

Importantly, elements of a rudimentary and functioning blood brain barrier (BBB) were also expressed in the wild type aiWBO simulations. In the AD simulations there were significant decreases in the astrocyte and pericyte components of the BBB. In addition, the function of the BBB was also significantly decreased in the AD simulations. The function of the BBB which was not explored in detail in these initial experiments, has important nutritional, metabolic and pharmacological implications which will be evaluated in futures experiments with a focus on drug development. The NEUBOrg platform aiWBO simulations should permit the early identification of drugs and other potential therapeutics which either enter or do not enter the central nervous system.

### Cellular Stresses and Neuron Cell Death

Our data suggested a significant up regulation of cellular stress as well as neuron cell death markers as shown in Figure 5A. These data suggest that our aiWBO- APOE4 (AD) simulations accurately reflected phenotypic features associated with AD in human brain.

Validated computer simulations would have several important advantages. These simulations can be customized and developed and deployed rapidly in a cost-effective manner when compared with wet lab organoid development. We are currently focused on addressing the increased cellular stresses and neural cell death that are seen in *in vitro* and now in simulated brain organoids. It is likely that these significant issues are the result of the lack of cardiovascular, pulmonary and renal systems. It should be faster and easier to optimize organoid cellular stress and neuronal death by modifying the simulated environment as required. We believe that the data presented here show the promising potential of aiWBO simulations to overcome the current limitation of developing wet lab brain organoid.

### Limitations and Promises of the Current aiWBO Simulations

As we continue to develop NEUBOrg simulation, a key limitation of this technology is the degree to which the NEUBOrg platform can accurately represent currently available whole brain organoids. These initial data confirm that the platform can accurately recreate a diverse range of previously unseen features and profiles attributed to human brain organoids in the published literature. Currently, any simulation of a biological system like the human brain will be less complex than the organ itself. To complicate this further, our understanding of the human brain is itself incomplete. Going forward, the key to evaluating the utility of the NEUBOrg platform will be obtaining new data for learning and from ongoing validation.

The central issue of incomplete learning data continues to improve on an almost daily basis. Version 5.0 contained 4,206 gene/proteins or phenotypic concepts and 37,223 non-zero relationships while the current version (6.1) contains 4,516 gene/proteins or phenotypic concepts and 41,493 non-zero relationships. Overall, the data in v6.1 represents more than 20% of the human genome compared with ∼18% in version 5.0. Included in this number is 4,270 new relationships specifically relevant to whole brain organoid creation. In addition, each gene/protein and phenotypic concept in DeepNEU v6.1 now has on average >9 gene/protein or phenotypic inputs and outputs. Second, advanced computer modeling systems still require wet lab confirmation, and this continues to be important for DeepNEU v6.1 as well. A major goal of this project was to make these findings regarding the potential research and therapeutic benefits of a validated whole lung simulation of Alzheimer’s Disease available to the global research community for wet lab disease modeling, drug discovery and repurposing at the very earliest opportunity. We also plan to continue to develop and validate these important simulations, while focusing on improving the microcirculation and BBB elements of aiWBOs. We are currently seeking development partners with the goal of empowering Alzheimer’s Disease research to better understand disease pathology, enable drug discovery and repurposing. We commit to making any additional information available at the earliest opportunity. Third, we have successfully migrated the upgraded DeepNEU platform to the IBM cloud. This emerging technology will permit more rapid organoid simulation development, disease modeling, therapeutic target identification, and drug repurposing for Alzheimer’s and other diseases. Finally, our technology continues to evolve from a hybrid deep learning approach toward the Wise Learning (WL) approach described by Groumpos (2016).

## Conclusion/Future Directions

With the current advances in brain organoid and iPSC-derived brain cells research, the functions of AD risk genes and AD causing mutations in iPSC-derived brain cell types remains largely unexplored. It is important to further our understanding of how such mutations, either alone or in in combination, affect the interactions between the different cell types of the brain is contributing to AD pathogenesis. We conclude that based on data presented here and the continued development of the NEUBOrg platform, our aiWBO simulations will not only contribute to enhancing our knowledge of AD pathogenesis, but also can be expanded to study different neurodegenerative diseases. NEUBOrg holds considerable promise for allowing us to answer previously unanswered questions, and ultimately to identify and implement effective treatments for AD and other diseases that primarily affect the central nervous system.

## Data Availability Statement

All datasets generated for this study are included in the article/Supplementary Material, further inquiries can be directed to the corresponding author/s.

## Author Contributions

SE: conceptualization, experimental work analysis, manuscript writing, and figures preparation. WRD: conceptualization, experimental work analysis, manuscript writing, figures preparation, performing all computational simulations, and AD disease modeling. Both authors contributed to the article and approved the submitted version.

## Conflict of Interest

SE and WRD have uncompensated relationships with 123 Genetix medical enterprise, nonetheless the authors declare that they are providing an unbiased scientific article.
