# Supplementary material for: NEUBOrg: Artificially Induced Pluripotent Stem Cell-Derived Brain Organoid to Model and Study Genetics of Alzheimer’s Disease Progression
Source: Front Aging Neurosci. 2021 Feb 23;13:643889. doi: 10.3389/fnagi.2021.643889 (PMC7940675; doi:10.3389/fnagi.2021.643889)
Supplement: Supplementary file 1 [file Data_Sheet_1.docx]

**Supplementary information**

Table S1: aiWBO main cell types simulated

| Cell types | Markers |
| --- | --- |
| Neurons (N=6) | DCX/Doublecortin, GAP43, MAP2, NeuN/RBFOX3, SLC17A7/VGLUT1, TUBB3 |
| Astrocytes (N=7) | CNTF, GFAP, GLAST-1/EAAT1, GLT-1/EAAT2, Neurotropin3, NGF, S100B |
| Oligodendrocytes (N=12) | miR-212 (-), Oligodendrocyte Apoptosis (-), BDNF (-), CNTF, Fibrinogen, LNRGF, Neurotropin3, NGF, Olig2, OPC-Differentiation, PDGF-R-alpha, Sox9 |
| Microglia (N=4) | AIF, CCL-4/MIP-1beta, CD11B/ITGAM, Neurotropin3 |
| Endothelial cells (N=3) | Mesoderm, VEGF, PIAS1/2 |
| Pericytes (N=5) | Amyloid-Beta-Oligomers/aggregation (-),  ETaR (-), PDGFR, TGFbR1/ALK5, TLR2/4 |

Table S2: aiWBO main Rostral Caudal Brain Regions simulated

| Rostral Caudal Brain Regions | Markers |
| --- | --- |
| Forebrain (N=11) | Emx1, FOXG1, GBX2, HOXA2, MAP2, NGF, Otx2, PAX6, Six3, Tbr-2/Tbr2, TUBB3 |
| Midbrain (N=16) | GBX2, HOXA2, DOPA decarboxylase, Engrailed-1&2, FOXA2, HOXA3, HOXB3, Irx1/2, Lmx1a, Lmx1b, Otx2, PAX3, PAX5, PAX7, PITX3, Tyrosine hydroxylase/TH |
| Hindbrain (N=10) | Irx3, EGR2/Krox20, GBX2, HOXA4, HOXB1, ISL1/Isl-1, LHX2/LH2, Lmx1b, PAX2, PAX5 |

Table S3: aiWBO main Ventral Dorsal Brain Regions simulated

| Ventral Dorsal Brain Regions | Markers |
| --- | --- |
| Ventral Forebrain | Dlx1/2, FOXG1, GSX2/GSH2, LHX2/LH2, LHX6, NKX-2.1/TITF1, Olig2, Otx2, Six3 |
| Pontine Nuclei (Ventral) | PCSK9 |
| Inferior Olive Complex (Ventral) | Deep Cerebellar Nuclei (-), Calbindin/CALB1, VGLUT2 |
| Cerebellum (Dorsal Hindbrain) | Olig2, GAD65, ATOH1, Tbr-1, VGLUT1 |
| Choroid Plexus (Dorsal Forebrain) | AQP1, En-1&2/Engrailed-1&2, Epithelial Cells, GBX2, PAX2, RSPO2, TTR/transthyretin |
| Deep Cerebellar Nuclei (Dorsal) | Cerebellum-PurL (-), Climbing Fibers, InfOlivaryCplx, Mossy Fibers, Pontine Nucleus |
| Thalamus (Dorsal Forebrain) | Calbindin/CALB1, Calretinin/CALB2, Parvalbumin (PV) |
| Hippocampus (Ventral and Dorsal) | FZD9, PROX1, GABARb, SLC17A7/VGLUT1 |

Table S4: aiWBO main Cerebral Cortical Layers simulated

| Cerebral Cortical Layers (outer to innermost) | Markers |
| --- | --- |
| Layer 1 (N=5) | Calbindin/CALB1, Reelin, Integrin-B1, nNOS/NOS1, Tbr-1/Tbr1 |
| Layer 2/3 (N=13) | Alzheimer's Disease (AD) (-), Neurofibrillary Tangles/(NFTs) (-), Calbindin/CALB1, Calretinin/Calb2, NEFH/N200, Reelin, BRN-2/POU3F2, CUX1, LMO4, MAP1b, PCP-2, SATB2, Tbr-1/Tbr1 |
| Layer 4 (N=7) | SATB2, Reelin, CUX1, MAP1b, NECAB1/STIP1, RORbeta, Tbr-1/Tbr1 |
| Layer 5 (N=16) | Neurofibrillary Tangles/(NFTs) (-), BRN-2/POU3F2, Calbindin/CALB1, Calretinin/CALB2, FOXP2, LMO4, TLE4, Reelin, FezF2, SATB2, Ctip2/BCL11B, ETV1/ER81, FOXO1/3, NEFH/N200, Sox5, Tbr-1/Tbr1 |
| Layer 6 (N=13) | ETV1/ER81, FezF2, SATB2, Reelin, Ctip2/BCL11B, DARPP-32, FOXP2, MAP1b, NEFH/N200, PCP-2, Sox5, Tbr-1/Tbr1, TLE4 |

Table S5: aiWBO main Cerebellar Cortical Layers simulated

| Cerebellar Cortical Layers (outer to innermost) | Markers |
| --- | --- |
| Layer 1 (Molecular Layer) (N=2) | GAD67, MX1 |
| Layer 2 (Purkinje Layer) (N=11) | Cerebellum-MolL-StC (-), Cerebellum-PurL-BasC (-), Calbindin/CALB1, GAD67, Astrocytes, Cerebellum-MolL-PFC, Climbing Fibers, MX1, Myosin IIb/MYH10, Parvalbumin (PV), PCP-2 |
| Layer 3 (Granular Layer) (N=5) | Cerebellum-GranL-GolC (-), Mossy Fibers, Neurons, NeuN/RBFOX3, NeuroD1 |
| Layer 4 (White Layer) (N=4) | Oligodendrocytes, MAP2K6/MEK6, Parvalbumin (PV), Plp1 |

Table S6: Effect on synaptic markers of synaptogenesis and function

| Synaptic Marker | WT-Average | WT-±95%CI | APOE4-Average | APOE4-±95%CI | p value |
| --- | --- | --- | --- | --- | --- |
| Homer | 0.042 | 0.002 | 0.018 | 0.005 | <0.001 |
| PSD95 | 0.969 | 0.005 | 0.694 | 0.204 | <0.05 |
| SLC17A7/VGLUT1 | -0.026 | 0.037 | -0.110 | 0.013 | <0.01 |
| SYN-1/Synapsin1 | 0.968 | 0.002 | 0.976 | 0.000 | <0.001 |
| Synaptic dysfunction | -0.998 | 0.001 | 0.696 | 0.051 | <0.001 |
| Synaptic plasticity | 1.000 | 0.000 | 0.999 | 0.000 | <0.001 |
| Synaptogenesis | 0.828 | 0.026 | 0.117 | 0.045 | <0.001 |

Appendix 1: Transparency statement about how 123Genetix creates artificially induced pluripotent stem cell (aiPSC) simulations

The DeepNEU platform (v. 6.1)

We have developed a novel hybrid deep-machine learning platform employing a fully connected recurrent neural network (RNN) like architecture in which each of the inputs is connected to its output nodes (feedforward neurons) and each of the output nodes is also connected back to their input nodes (feedback neurons). There are at least two major benefits of using this network architecture. First, RNN can use the feedback neurons connections to store information over time and develop “memory”. Second, RNN networks can handle sequential data of arbitrary length [1]. For example, RNN can be programmed to simulate the relationship of a specific gene/protein/phenotypic concept to another gene/protein/phenotypic concept (one to one), gene/protein/phenotypic concept to multiple genes/proteins/phenotypic concepts (one to many), multiple genes/proteins/phenotypic concepts to one gene/protein/phenotypic concept (many to one) and multiple genes/proteins/phenotypic concepts to different multiple genes/proteins/phenotypic concepts (many to many). Our RNN like DeepNEU network was developed with one network processing layer for each input to promote complex learning and analysis of how different genes and pathways are potentially regulated in embryonic and reprogrammed somatic cells in key signaling pathways. Here we have used the DeepNEU platform to simulate aiPSCs by using defined sets of reprogramming factors (genes/proteins were turned on or off based on the modeled iPSCs).

The Dataset

We have incorporated into the DeepNEU database key genes/proteins/phenotypic concepts that were reported to be involved in regulating and maintaining signaling pathways in human embryonic stem cells (hESCs) and induced human pluripotent stem cells (hiPSCs). We have gathered genes/proteins/phenotypic concepts based on literature reports that extensively studied cellular pathways of hESC and/or hiPSC [2–11]. Abundant data were available. As of this writing, a PubMed Central (PMC) search of the literature with “stem cells” returned more than 697,000 hits. A more focused query using “stem cell signaling”, returned more than 405,600,020 hits. The data that were included in the DeePNEU database were selected with a preference for (1) human stem cell data, (2) recency of peer reviewed English language publications and (3) highest impact factors of the journals under consideration. To that end, the data was used to create a database of important genes/proteins/phenotypic concepts and relationships based on the documented contributions to human stem cell signaling pathways. The current version of the database (v. 6.1) includes 4516 genes/proteins/phenotypic concepts (inputs) involved in hESC cellular pathways and 41,493 gene/protein/phenotypic relationships important in hESC that were used for aiPSC system modelling. Notably, this simple data representation permits complex relationships including both positive and negative feedback loops that are common in biological systems.

All data (genes/proteins/phenotypic concepts, and relationships) were entered, formatted and stored as a large CSV (comma separated values) file in Delimit Professional (v4.1.3, Delimitware, 2020). This database manager was chosen because it can efficiently handle very large CSV files where data can be represented as an NxN (an array of values with N rows and N columns) relationship matrix. In addition, built-in data entry and file scan functions help to ensure and maintain data integrity. This software can also import and export multiple data file types facilitating two-way interaction with a wide range of data analysis tools. Finally, the software scales easily to NxN or NxM (an array of values with N rows and M columns) databases having millions of rows and columns (www.delimitware.com, 2020).

The DeepNEU Logic

The DeepNEU platform uses a novel, but powerful neutrosophic logical (NL) framework to represent relationships between signaling genes/proteins/phenotypic concepts. NL was originally described by Florentin Smarandache in 1995. In NL, every logical variable X is described by an ordered triple, X = (T, I, F) where T is the degree of truth, “I” is the degree of indeterminacy, and F is the degree of falseness. The strength of any relationship can have any real value between − 1 and + 1 or “I” if the relationship is considered indeterminate. Positive or stimulatory causal relationships are represented by + 1 in the database unless there is a fractional value > 0 and < + 1. Similarly, negative or inhibitory causal relationships are represented by − 1 in the database unless a fractional value < 0 and > − 1 is provided. Relationships are considered indeterminate and represented by an “I” if multiple sources report conflicting data or if the relationship is labelled with a question mark in an associated process flow diagram. A value of zero is used when no relationship between nodes is known or suspected [12]. Importantly, NL is an extension and generalization of Fuzzy Logic (FL) and can be easily converted to FL by replacing all indeterminate (I) relationships with zeros (i.e. by assuming there is no causal relationship).

DeepNEU network architecture

The NxN relationship matrix is the core data for the unsupervised fully connected RNN like simulations. A learning system is referred to as supervised when each data pattern is associated with a specific numerical (i.e., regression) or category (i.e., classification) outcome. Unsupervised learning is used to draw inferences from datasets consisting of input data patterns that do not have labeled outcomes [12]. DeepNEU is a complex learning system in that every (gene/protein/phenotypic concept) node in the multilayered network is connected to every other node in the network. Traditional neural networks have one or a few hidden or processing layers between the input layer and the output layer. Advanced deep-learning neural networks can have more than a dozen processing layers [13,14]. DeepNEU has one processing layer for each input variable. Taken together, the input variables and the declared initial values constitute an N-dimensional initial input or state vector. Vector-Matrix multiplication uses this N-dimensional input vector and the NxN relationship matrix to produce an N-dimensional output or new state vector. The new state vector becomes the new input vector for the next iteration and this iterative process continues until a new system wide steady state is achieved. In general terms, the DeepNEU network architecture is closely related to Neutrosophic and Fuzzy Cognitive Maps (NCMs/FCMs); used to represent causal relationship between concepts (i.e., genes/proteins/phenotypic) which are also examples of fully connected and recurrent neural networks [15, 16].

The DeepNEU simulations

The goal of the initial project was to first create a computer simulation of a hiPSC and then validate the model using the results published by Takahashi et al. in 2007 and others as described above. Briefly, the input or initial state vector of dimension N was set to all zeros except for transcription factors OCT3/4, SOX2, KLF4 and cMYC. These four factors were given a value of + 1 indicating that they were turned on for the first iteration. These values were not locked on so that all subsequent values were determined by system behavior.

DeepNEU simulation protocol

1. The machine learning process began with vector matrix multiplication (VMM). The NxN relationship matrix was multiplied by the “N”- dimensioned input vector with OCT3/4, SOX2, KLF4 and cMYC turned on. Both the input vector and relationship matrix are comprised mostly of zeros. The input vector and relationship matrix were both considered to be sparse. To minimize the computational burden, sparse vector matrix multiplication algorithms were employed at each iteration during model generation.

2. At each iteration the sparse VMM operation produces an “N”-dimensional output vector with variable components many of which have large positive or negative values. To avoid computational explosion a squashing or activation function was used to map these values between a minimum of − 1 and a maximum of + 1. After initial evaluation of several activation functions, a sigmoid variant function was selected based on rapidity of system convergence and outcome reproducibility.. At the end of the activation process, the squashed N-dimensional output vector becomes the new input vector for the next iteration. This cycle is repeated until system convergence occurs indicating that a new system wide steady state has been achieved.

3. The goal of the learning system is to minimize error. In this case the error being considered is the mean squared error (MSE) between a given output vector and the previous output vector. During model development several error functions including adjusted R2, SVM/Vapnik loss and MSE were evaluated. The MSE function was selected because its’ use consistently resulted in faster system convergence and more reproducible results. While the MSE function has been widely used it has also been widely criticized because the function can perform poorly due to squaring in the presence of outliers. In the current project, the error function was applied after the raw system output was “squashed” between values of − 1 and + 1 using a sigmoid type function. This squashing effectively mitigates the problem of potential outliers. As learning continues the MSE converges towards zero. For this project system convergence was defined at MSE < 0.001 and model generation stops. The system output is then saved as a CSV data file for further analysis.

4. The final output from the aiPSC model regarding the expression or repression of genes and proteins was directly compared with published expression profiles [18]. Model prediction values > 0 were classified as expressed or upregulated while values < 0 were classified as not expressed or downregulated. Statistical analysis of the aiPSC predictions and the published data used the binomial test. This test was selected because it provides an exact probability, can compensate for prediction bias and is ideal for determining the statistical significance of experimental deviations from an actual distribution of observations that fall into two outcome categories (e.g., agree vs disagree). A p-value < 0.05 is considered significant and is interpreted to indicate that the observed relationship between aiPSC predictions and actual outcomes is unlikely to have occurred by chance alone.

Once the aiPSC simulations were validated against available peer reviewed wet lab results, the aiPSC simulations were further developed to create simulations of a whole brain organoid as outlined in the METHODS section of the paper.

References for Appendix 1

1. Lipton ZC, Berkowitz J, Elkan C. A critical review of recurrent neural

networks for sequence learning. arXiv preprint arXiv:150600019; 2015.

2. Aggarwal S, Pittenger MF. Human mesenchymal stem cells modulate

allogeneic immune cell responses. Blood. 2005;105:1815–22.

3. Ben-Porath I, Thomson MW, Carey VJ, Ge R, Bell GW, Regev A, Weinberg RA.

An embryonic stem cell–like gene expression signature in poorly

differentiated aggressive human tumors. Nat Genet. 2008;40:499.

4. Bernstein BE, Mikkelsen TS, Xie X, Kamal M, Huebert DJ, Cuff J, Fry B,

Meissner A, Wernig M, Plath K. A bivalent chromatin structure marks key

developmental genes in embryonic stem cells. Cell. 2006;125:315–26.

5. Boyer LA, Lee TI, Cole MF, Johnstone SE, Levine SS, Zucker JP, Guenther MG,

Kumar RM, Murray HL, Jenner RG. Core transcriptional regulatory circuitry in

human embryonic stem cells. cell. 2005;122:947–56.

6. Hamazaki T, El Rouby N, Fredette NC, Santostefano KE, Terada N. Concise

review: induced pluripotent stem cell research in the era of precision

medicine. Stem Cells. 2017;35:545–50.

7. Odorico JS, Kaufman DS, Thomson JA. Multilineage differentiation from

human embryonic stem cell lines. Stem Cells. 2001;19:193–204.

8. Sato N, Meijer L, Skaltsounis L, Greengard P, Brivanlou AH. Maintenance of

pluripotency in human and mouse embryonic stem cells through activation

of Wnt signaling by a pharmacological GSK-3-specific inhibitor. Nat Med.

2004;10:55.

9. Tachibana M, Amato P, Sparman M, Gutierrez NM, Tippner-Hedges R, Ma H,

Kang E, Fulati A, Lee H-S, Sritanaudomchai H. Human embryonic stem cells

derived by somatic cell nuclear transfer. Cell. 2013;153:1228–38.

10. Vallier L, Alexander M, Pedersen RA. Activin/nodal and FGF pathways

cooperate to maintain pluripotency of human embryonic stem cells. J Cell

Sci. 2005;118:4495–509.

11. Zhao XD, Han X, Chew JL, Liu J, Chiu KP, Choo A, Orlov YL, Sung W-K,

Shahab A, Kuznetsov VA. Whole-genome mapping of histone H3 Lys4 and

27 trimethylations reveals distinct genomic compartments in human

embryonic stem cells. Cell Stem Cell. 2007;1:286–98.

12. Smarandache F. Neutrosophic logic-a generalization of the intuitionistic

fuzzy logic. Multispace & Multistructure Neutrosophic Transdisciplinarity (100

collected papers of Science). 2010;4:396.

13. Groumpos VP, Biniari K, Groumpos PP. A new mathematical modelling

approach for viticulture and winemaking using fuzzy cognitive maps. Paper

presented at: ELEKTRO, 2016 (IEEE); 2016.

14. Wang H, Raj B, Xing EP. On the origin of deep learning. arXiv preprint arXiv:

170207800; 2017.

15. Kandasamy WV, Smarandache F. Fuzzy cognitive maps and neutrosophic

cognitive maps (infinite study); 2003.

16. William MA, Devadoss AV, Sheeba JJ. A study on Neutrosophic cognitive

maps (NCMs) by analyzing the risk factors of breast Cancer. International

Journal of Scientific & Engineering Research. 2013;4:1–4.

18. Takahashi K, Tanabe K, Ohnuki M, Narita M, Ichisaka T, Tomoda K, Yamanaka

S. Induction of pluripotent stem cells from adult human fibroblasts by

defined factors. cell. 2007b;131:861–72.
